# 1-(1-Benzofuran-2-yl)-3-(4-chloro­phen­yl)prop-2-en-1-one

**DOI:** 10.1107/S1600536810003004

**Published:** 2010-01-30

**Authors:** S. Jeyaseelan, H. C. Devarajegowda, G. Venkatarama, M. Vinduvahini, Alphonsus D’souza

**Affiliations:** a Department of Physics, Yuvaraja’s College (Constituent College), University of Mysore, Mysore 570 005, Karnataka, India; b Department of Physics, Sri D. Devaraj urs. First Grade College, Hunsur 571 105, Karnataka, India; c Department of Chemistry, St. Philomena’s College, Mysore 570 015, Karnataka, India

## Abstract

In the title compound, C_17_H_11_ClO_2_, the benzofuran ring system is almost planar (r.m.s. deviation = 0.011 Å) and forms a dihedral angle of 10.53 (6)° with the chloro­phenyl ring. No significant inter­molecular inter­actions are observed.

## Related literature

For general background to chalcone, see: Dhar (1981 ▶). For the biological properties of benzofuran derivatives, see: Nasef *et al.* (1992 ▶); Bogolyubsakaya & Perovich (1964 ▶); Deshmukh *et al.* (2004 ▶); Stanislav *et al.* (2000 ▶); Brady *et al.* (1973 ▶); Kamal *et al.* (2006 ▶); Alejandro *et al.* (2008 ▶); Rajesh *et al.* (2006 ▶). For related structures, see: Devarajegowda *et al.* (2001 ▶); Kant *et al.* (2009 ▶).
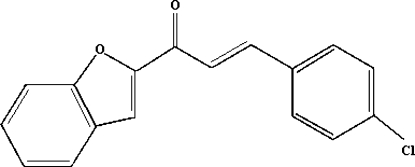


## Experimental

### 

#### Crystal data

C_17_H_11_ClO_2_
*M* *_r_* = 282.71
Monoclinic, 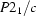

*a* = 15.9034 (12) Å
*b* = 14.1393 (12) Å
*c* = 5.9572 (5) Å
β = 93.039 (4)°
*V* = 1337.67 (19) Å^3^
*Z* = 4
Mo *K*α radiation
μ = 0.28 mm^−1^
*T* = 293 K
0.22 × 0.20 × 0.10 mm

#### Data collection

Bruker SMART CCD area-detector diffractometer
Absorption correction: multi-scan (*SADABS*; Sheldrick, 2004 ▶) *T*_min_ = 0.940, *T*_max_ = 0.972
12871 measured reflections
3323 independent reflections
2665 reflections with *I* > 2σ(*I*)
*R*_int_ = 0.026

#### Refinement

*R*[*F* ^2^ > 2σ(*F* ^2^)] = 0.040
*wR*(*F* ^2^) = 0.115
*S* = 1.02
3323 reflections
182 parameters
H-atom parameters constrained
Δρ_max_ = 0.21 e Å^−3^
Δρ_min_ = −0.24 e Å^−3^

### 

Data collection: *SMART* (Bruker, 2001 ▶); cell refinement: *SAINT* (Bruker, 2001 ▶); data reduction: *SAINT*; program(s) used to solve structure: *SHELXS97* (Sheldrick, 2008 ▶); program(s) used to refine structure: *SHELXL97* (Sheldrick, 2008 ▶); molecular graphics: *ORTEP-3* (Farrugia, 1997 ▶); software used to prepare material for publication: *SHELXL97*.

## Supplementary Material

Crystal structure: contains datablocks global, I. DOI: 10.1107/S1600536810003004/ci5024sup1.cif

Structure factors: contains datablocks I. DOI: 10.1107/S1600536810003004/ci5024Isup2.hkl

Additional supplementary materials:  crystallographic information; 3D view; checkCIF report

## Figures and Tables

**Fig. 1.**
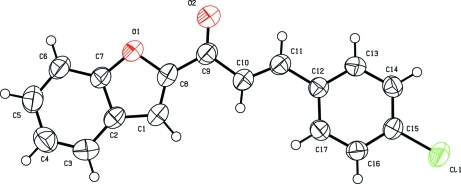
The molecular structure of the title compound. Displacement ellipsoids are drawn at the 50% probability level.

## supplementary crystallographic information

### Comment

Chalcones have been recognized as a significant field of study for a long time because of a variety of biological activities as well as they serve as intermediates in the synthesis of a variety of heterocyclic compounds. Several heterocyclic analogues of chalcones have been reported to possess antibacterial, bacteriostatic tuberculostatic, insecticidal, antiparasitic, coronary vasodilating and choleretic activities (Dhar, 1981). Further, benzofuran derivatives have been reported to possess sedative and hypnotic (Nasef *et al.*, 1992), antiinflammatory (Bogolyubsakaya & Perovich, 1964), antidepressant (Deshmukh *et al.*, 2004), analgesic (Stanislav *et al.*, 2000), hypoglycemic (Brady *et al.*, 1973), anticonvulsant (Kamal *et al.*, 2006), antibacterial (Alejandro *et al.*, 2008) and antifungal activities (Rajesh *et al.*, 2006). Owing to these biological activities of benzofuran propenones and in an attempt to study the structure-activity relationship of benzofuran and related systems, it was contemplated to synthesize benzofuryl propenones and the crystal structure of one of them, the title compound, is reported.

The benzofuran ring system is almost planar (r.m.s. deviation 0.011 Å). The dihedral angle between the benzofuran ring system and the chlorophenyl ring is 10.53 (6)°. Bond distances within the aromatic rings are in agreement with those observed in related structures (Devarajegowda *et al.*, 2001; Kant *et al.*, 2009).

### Experimental

A mixture of 2-acetylbenzofuran (0.01 mol) and p-chlorobenzaldehyde (0.01 mol) in ethanol (20 ml) was stirred for 24 h in aqueous NaOH (8 ml). It was then diluted with water (100 ml) and acidified with concentrated HCl. The course of the reaction was monitored by TLC using chloroform-carbon disulfide (1:1). The product obtained was filtered, washed with water and recrystallised from ethanol (m.p. 398 K). Spectral data IR (KBr) cm^-1^: 1650 (C═O), 1620 (C═C stretching in aromatic), 1080 (C-O-C of benzofuran). ^1^H NMR (CDCl_3_): 6.67-7.2 (m, 9H, Ar-H), 6.70 (d, 1H, -COCH=), 8.60 (d, 1H, =CH-Ar).

### Refinement

H atoms were positioned at calculated positions [C-H = 0.93 Å] and refined using a riding model with *U*_iso_(H) = 1.2*U*_eq_(C).

### Crystal data

**Table:**

C_17_H_11_ClO_2_	*F*(000) = 584
*M**_r_* = 282.71	*D*_x_ = 1.404 Mg m^−^^3^
Monoclinic, *P*2_1_/*c*	Melting point: 398 K
Hall symbol: -P 2ybc	Mo *K*α radiation, λ = 0.71073 Å
*a* = 15.9034 (12) Å	Cell parameters from 3323 reflections
*b* = 14.1393 (12) Å	θ = 1.3–28.4°
*c* = 5.9572 (5) Å	µ = 0.28 mm^−^^1^
β = 93.039 (4)°	*T* = 293 K
*V* = 1337.67 (19) Å^3^	Plate, white
*Z* = 4	0.22 × 0.20 × 0.10 mm

### Data collection

**Table:**

Bruker SMART CCD area-detector diffractometer	3323 independent reflections
Radiation source: fine-focus sealed tube	2665 reflections with *I* > 2σ(*I*)
graphite	*R*_int_ = 0.026
ω and φ scans	θ_max_ = 28.4°, θ_min_ = 1.3°
Absorption correction: multi-scan (*SADABS*; Sheldrick, 2004)	*h* = −21→20
*T*_min_ = 0.940, *T*_max_ = 0.972	*k* = −18→18
12871 measured reflections	*l* = −7→7

### Refinement

**Table:**

Refinement on *F*^2^	Secondary atom site location: difference Fourier map
Least-squares matrix: full	Hydrogen site location: inferred from neighbouring sites
*R*[*F*^2^ > 2σ(*F*^2^)] = 0.040	H-atom parameters constrained
*wR*(*F*^2^) = 0.115	*w* = 1/[σ^2^(*F*_o_^2^) + (0.0527*P*)^2^ + 0.3221*P*] where *P* = (*F*_o_^2^ + 2*F*_c_^2^)/3
*S* = 1.02	(Δ/σ)_max_ = 0.001
3323 reflections	Δρ_max_ = 0.21 e Å^−^^3^
182 parameters	Δρ_min_ = −0.24 e Å^−^^3^
0 restraints	Extinction correction: *SHELXL97* (Sheldrick, 2008), Fc^*^=kFc[1+0.001xFc^2^λ^3^/sin(2θ)]^-1/4^
Primary atom site location: structure-invariant direct methods	Extinction coefficient: 0.0117 (16)

### Special details

**Table:**

Geometry. All esds (except the esd in the dihedral angle between two l.s. planes) are estimated using the full covariance matrix. The cell esds are taken into account individually in the estimation of esds in distances, angles and torsion angles; correlations between esds in cell parameters are only used when they are defined by crystal symmetry. An approximate (isotropic) treatment of cell esds is used for estimating esds involving l.s. planes.
Refinement. Refinement of F^2^ against ALL reflections. The weighted R-factor wR and goodness of fit S are based on F^2^, conventional R-factors R are based on F, with F set to zero for negative F^2^. The threshold expression of F^2^ > 2sigma(F^2^) is used only for calculating R-factors(gt) etc. and is not relevant to the choice of reflections for refinement. R-factors based on F^2^ are statistically about twice as large as those based on F, and R- factors based on ALL data will be even larger.

### Fractional atomic coordinates and isotropic or equivalent isotropic displacement parameters (Å^2^)

**Table:**

	*x*	*y*	*z*	*U*_iso_*/*U*_eq_	
C1	1.22464 (9)	0.64723 (11)	0.5466 (3)	0.0508 (4)	
H1	1.1909	0.6671	0.4232	0.061*	
C2	1.31402 (10)	0.64001 (11)	0.5583 (2)	0.0497 (3)	
C3	1.37837 (12)	0.65858 (13)	0.4117 (3)	0.0628 (4)	
H3	1.3660	0.6820	0.2679	0.075*	
C4	1.45972 (12)	0.64125 (15)	0.4865 (3)	0.0717 (5)	
H4	1.5031	0.6535	0.3920	0.086*	
C5	1.47890 (11)	0.60577 (15)	0.7004 (3)	0.0692 (5)	
H5	1.5349	0.5939	0.7447	0.083*	
C6	1.41797 (10)	0.58781 (13)	0.8476 (3)	0.0595 (4)	
H6	1.4311	0.5644	0.9911	0.071*	
C7	1.33559 (9)	0.60625 (11)	0.7727 (2)	0.0485 (3)	
C8	1.19823 (9)	0.62016 (11)	0.7456 (3)	0.0517 (4)	
C9	1.11355 (10)	0.61170 (12)	0.8310 (3)	0.0540 (4)	
C10	1.04368 (10)	0.62421 (13)	0.6625 (3)	0.0560 (4)	
H10	1.0546	0.6508	0.5242	0.067*	
C11	0.96572 (9)	0.59891 (11)	0.7013 (3)	0.0493 (3)	
H11	0.9572	0.5719	0.8407	0.059*	
C12	0.89149 (8)	0.60906 (10)	0.5471 (2)	0.0440 (3)	
C13	0.81273 (9)	0.58296 (11)	0.6193 (2)	0.0480 (3)	
H13	0.8090	0.5549	0.7596	0.058*	
C14	0.74015 (9)	0.59803 (11)	0.4862 (3)	0.0501 (3)	
H14	0.6879	0.5812	0.5368	0.060*	
C15	0.74645 (9)	0.63839 (11)	0.2773 (2)	0.0473 (3)	
C16	0.82376 (9)	0.66272 (11)	0.1978 (2)	0.0493 (3)	
H16	0.8271	0.6888	0.0553	0.059*	
C17	0.89576 (9)	0.64786 (11)	0.3322 (2)	0.0489 (3)	
H17	0.9479	0.6638	0.2792	0.059*	
O1	1.26490 (6)	0.59456 (8)	0.89122 (17)	0.0552 (3)	
O2	1.10294 (8)	0.59470 (11)	1.0286 (2)	0.0755 (4)	
Cl1	0.65624 (3)	0.66004 (4)	0.10847 (8)	0.06940 (17)	

### Atomic displacement parameters (Å^2^)

**Table:**

	*U*^11^	*U*^22^	*U*^33^	*U*^12^	*U*^13^	*U*^23^
C1	0.0515 (8)	0.0488 (8)	0.0506 (8)	0.0031 (6)	−0.0110 (6)	0.0007 (6)
C2	0.0542 (8)	0.0455 (8)	0.0481 (8)	0.0008 (6)	−0.0081 (6)	−0.0025 (6)
C3	0.0775 (11)	0.0621 (10)	0.0489 (8)	0.0026 (8)	0.0042 (8)	0.0027 (7)
C4	0.0633 (11)	0.0824 (13)	0.0706 (11)	−0.0021 (9)	0.0149 (9)	−0.0053 (10)
C5	0.0491 (8)	0.0854 (13)	0.0723 (11)	0.0035 (8)	−0.0041 (8)	−0.0112 (10)
C6	0.0537 (8)	0.0719 (11)	0.0515 (8)	0.0067 (8)	−0.0108 (7)	−0.0022 (8)
C7	0.0486 (7)	0.0495 (8)	0.0466 (7)	−0.0014 (6)	−0.0050 (6)	−0.0008 (6)
C8	0.0480 (7)	0.0488 (8)	0.0567 (8)	0.0013 (6)	−0.0114 (6)	−0.0001 (7)
C9	0.0519 (8)	0.0550 (9)	0.0541 (9)	−0.0010 (7)	−0.0061 (6)	0.0060 (7)
C10	0.0483 (8)	0.0646 (10)	0.0544 (9)	0.0013 (7)	−0.0038 (6)	0.0095 (7)
C11	0.0496 (7)	0.0517 (8)	0.0462 (7)	0.0034 (6)	−0.0015 (6)	0.0019 (6)
C12	0.0445 (7)	0.0425 (7)	0.0450 (7)	0.0025 (6)	0.0014 (5)	−0.0011 (6)
C13	0.0502 (7)	0.0496 (8)	0.0444 (7)	−0.0016 (6)	0.0041 (6)	0.0045 (6)
C14	0.0442 (7)	0.0533 (9)	0.0530 (8)	−0.0057 (6)	0.0052 (6)	0.0002 (7)
C15	0.0456 (7)	0.0467 (8)	0.0490 (7)	−0.0011 (6)	−0.0042 (6)	−0.0034 (6)
C16	0.0529 (8)	0.0536 (8)	0.0414 (7)	−0.0046 (6)	0.0013 (6)	0.0026 (6)
C17	0.0444 (7)	0.0559 (9)	0.0468 (7)	−0.0025 (6)	0.0061 (6)	−0.0008 (6)
O1	0.0499 (6)	0.0655 (7)	0.0491 (6)	0.0006 (5)	−0.0073 (4)	0.0076 (5)
O2	0.0602 (7)	0.1083 (11)	0.0573 (7)	−0.0042 (7)	−0.0050 (5)	0.0175 (7)
Cl1	0.0526 (2)	0.0844 (3)	0.0690 (3)	−0.00645 (19)	−0.01679 (19)	0.0098 (2)

### Geometric parameters (Å, °)

**Table:**

C1—C8	1.335 (2)	C9—C10	1.468 (2)
C1—C2	1.423 (2)	C10—C11	1.323 (2)
C1—H1	0.93	C10—H10	0.93
C2—C7	1.389 (2)	C11—C12	1.4641 (19)
C2—C3	1.405 (2)	C11—H11	0.93
C3—C4	1.368 (3)	C12—C13	1.3954 (19)
C3—H3	0.93	C12—C17	1.398 (2)
C4—C5	1.388 (3)	C13—C14	1.382 (2)
C4—H4	0.93	C13—H13	0.93
C5—C6	1.365 (3)	C14—C15	1.377 (2)
C5—H5	0.93	C14—H14	0.93
C6—C7	1.386 (2)	C15—C16	1.384 (2)
C6—H6	0.93	C15—Cl1	1.7352 (15)
C7—O1	1.3690 (18)	C16—C17	1.378 (2)
C8—O1	1.3820 (17)	C16—H16	0.93
C8—C9	1.469 (2)	C17—H17	0.93
C9—O2	1.2216 (19)		
			
C8—C1—C2	107.24 (13)	C10—C9—C8	115.33 (14)
C8—C1—H1	126.4	C11—C10—C9	122.03 (15)
C2—C1—H1	126.4	C11—C10—H10	119.0
C7—C2—C3	118.89 (14)	C9—C10—H10	119.0
C7—C2—C1	105.49 (14)	C10—C11—C12	126.58 (15)
C3—C2—C1	135.61 (15)	C10—C11—H11	116.7
C4—C3—C2	118.14 (16)	C12—C11—H11	116.7
C4—C3—H3	120.9	C13—C12—C17	118.30 (13)
C2—C3—H3	120.9	C13—C12—C11	119.20 (13)
C3—C4—C5	121.44 (17)	C17—C12—C11	122.45 (13)
C3—C4—H4	119.3	C14—C13—C12	121.23 (13)
C5—C4—H4	119.3	C14—C13—H13	119.4
C6—C5—C4	121.88 (17)	C12—C13—H13	119.4
C6—C5—H5	119.1	C15—C14—C13	118.93 (13)
C4—C5—H5	119.1	C15—C14—H14	120.5
C5—C6—C7	116.70 (16)	C13—C14—H14	120.5
C5—C6—H6	121.7	C14—C15—C16	121.37 (13)
C7—C6—H6	121.7	C14—C15—Cl1	119.95 (12)
O1—C7—C6	126.81 (14)	C16—C15—Cl1	118.68 (11)
O1—C7—C2	110.25 (12)	C17—C16—C15	119.29 (13)
C6—C7—C2	122.94 (15)	C17—C16—H16	120.4
C1—C8—O1	111.44 (14)	C15—C16—H16	120.4
C1—C8—C9	131.92 (14)	C16—C17—C12	120.83 (13)
O1—C8—C9	116.62 (14)	C16—C17—H17	119.6
O2—C9—C10	122.98 (15)	C12—C17—H17	119.6
O2—C9—C8	121.69 (14)	C7—O1—C8	105.57 (12)
			
C8—C1—C2—C7	0.86 (17)	O2—C9—C10—C11	−14.3 (3)
C8—C1—C2—C3	−178.91 (18)	C8—C9—C10—C11	165.10 (16)
C7—C2—C3—C4	0.8 (2)	C9—C10—C11—C12	179.27 (15)
C1—C2—C3—C4	−179.41 (18)	C10—C11—C12—C13	−176.76 (16)
C2—C3—C4—C5	0.4 (3)	C10—C11—C12—C17	0.4 (3)
C3—C4—C5—C6	−1.0 (3)	C17—C12—C13—C14	−2.4 (2)
C4—C5—C6—C7	0.4 (3)	C11—C12—C13—C14	174.96 (14)
C5—C6—C7—O1	−179.32 (16)	C12—C13—C14—C15	0.9 (2)
C5—C6—C7—C2	0.9 (3)	C13—C14—C15—C16	0.9 (2)
C3—C2—C7—O1	178.67 (14)	C13—C14—C15—Cl1	−178.88 (12)
C1—C2—C7—O1	−1.15 (17)	C14—C15—C16—C17	−1.2 (2)
C3—C2—C7—C6	−1.5 (2)	Cl1—C15—C16—C17	178.57 (12)
C1—C2—C7—C6	178.64 (15)	C15—C16—C17—C12	−0.3 (2)
C2—C1—C8—O1	−0.28 (18)	C13—C12—C17—C16	2.0 (2)
C2—C1—C8—C9	−179.04 (16)	C11—C12—C17—C16	−175.20 (14)
C1—C8—C9—O2	−172.20 (18)	C6—C7—O1—C8	−178.80 (16)
O1—C8—C9—O2	9.1 (2)	C2—C7—O1—C8	0.98 (16)
C1—C8—C9—C10	8.3 (3)	C1—C8—O1—C7	−0.42 (17)
O1—C8—C9—C10	−170.36 (14)	C9—C8—O1—C7	178.55 (13)
